# Eco-friendly synthesis of NiO and Ag/NiO nanoparticles: applications in photocatalytic and antibacterial activities

**DOI:** 10.1098/rsos.241733

**Published:** 2025-02-12

**Authors:** T. N. Ravishankar, A. Ananda, B. M. Shilpa, J. R. Adarsh

**Affiliations:** ^1^ Department of Chemistry, B.M.S. College of Engineering, Bull Temple Road, Basavanagudi, Bengaluru, Karnataka 560019, India; ^2^ Department of Chemistry, Dayananda Sagar Academy of Technology & Management Udayapura, Kanakapura Road, Bengaluru 560082, India; ^3^ Department of Psychology, Christ University, Bangalore Kengeri Campus, Bangalore, Karnataka 560074, India; ^4^ Department of Chemistry, Global Academy of Technology, RR Nagar, Bangalore, Karnataka 560098, India

**Keywords:** NiO NPs, Ag/NiO NPs, *Cocos nucifera*, dye, photocatalysis, H_2_ production

## Abstract

Herein, NiO and Ag/NiO NPs were produced via the solution combustion method using nickel nitrate and silver nitrate as oxidizers and *Cocos nucifera* water as a fuel at 450°C. The study also explores their applications in photocatalytic dye degradation, H_2_ production and antibacterial properties. The primary advantage of using *C. nucifera* water as a green fuel in the solution combustion method is that it serves a dual purpose—both as a fuel and as a solvent. *This eliminates the need for additional water to create a homogeneous redox mixture of fuel and oxidant in the experimental procedure*. X-ray diffraction confirmed the existence of Ag in the bunsenite form of rhombohedral structure with a simple cubic system, with particles sized at 31–44 nm. Energy-dispersive X-ray spectroscopy revealed Ni, O and Ag weight percentages of 48.2, 44.5 and 7.3%, respectively. X-ray photoelectron spectroscopy confirmed the formation of Ag in NiO nanostructure. UV–visible spectrometry showed reduced band gap energy of Ag/NiO NPs (3.03–2.87 eV) compared to the bare NiO NPs (3.21 eV), red shift of the optical response towards the visible region after doping Ag into the NiO. The 0.3 wt% Ag/NiO NPs showed the highest quantum efficiency (0.781) among the other synthesized NPs. Fourier-transform infrared spectroscopy revealed absorption bands in the range of 460–900 cm^−1^ stretching vibrations of Ni–O and Ag–O. Photoluminescence spectroscopy indicated that a doping concentration of 0.3 wt% Ag effectively introduces donor levels, defect levels and surface trap states within the NiO nanocrystalline structure, enhancing charge carrier separation and reducing recombination. Scanning electron microscopy revealed a voluminous, porous surface morphology characterized by numerous voids, resulting from the release of various combustible gases during the combustion process. Transmission electron microscopy images showed that most particles were spherical, irregular in size and well-distributed, with minimal aggregation with an average particle size of 25.8 nm. BET analysis of both NiO and 0.3 wt% Ag/NiO NPs exhibited type IV adsorption isotherms, indicating mesoporous structures and a clear monolayer–multilayer adsorption process, 0.3 wt% Ag/NiO NPs showed the highest surface area (170 m^2^ g^−1^) compared to the NiO (130 m^2^ g^−1^) NPs. Ag/NiO NPs has demonstrated a promising H_2_ evolution rate of 1212 μmol g⁻¹ under visible light illumination in a water/ethanol system. The trypan blue dye degradation reaches up to 98% and has moderate stability for the reusable photocatalysis process. The synthesized NPs exhibited significantly enhanced antibacterial activity against a range of bacterial strains.

## 1. Introduction

Transition metal oxide nanoparticles (NPs) have garnered significant attention due to their enhanced physical and chemical properties compared to bulk materials [1,2]. These improved properties have led to their extensive use in a variety of fields, including photocatalysis [3], battery technology [4], sensing materials [5], photovoltaic cells [6], supercapacitors [7], medical applications, preservation of fruits [8] and others. Among different transition metal oxide NPs, NiO NPs are particularly noteworthy due to their exceptional chemical and thermal stability. They also show significant promise for applications in energy production and storage devices [9,10]. Various research groups have demonstrated that bare NiO NPs exhibit limited photocatalytic activity due to slow electron transfer to oxygen and a high rate of recombination of excited electron–hole pairs [11–13]. To address these limitations, researchers worldwide synthesized noble metal-doped NiO NPs to enhance their photocatalytic performance [14,15]. Among various noble metals, silver (Ag) is regarded as one of the most effective doping agents [16,17]. It modifies the surface properties and nanocrystalline structure of NiO NPs, reduces the recombination rate of electron–hole pairs and increases the availability of charge carriers for redox reactions on the surface of the NiO NPs [18,19]. Several methods are available for preparing NiO and Ag/NiO NPs, including hydrothermal, sol–gel, precipitation, chemical vapour deposition and solution combustion techniques, among others [20–22]. Solution combustion is an intriguing topic, essentially involving the propagation of self-sustained exothermic reactions in aqueous or sol–gel media. A variety of nanoscale materials, including oxides, composites, alloys, metals and sulfides, have been synthesized using the solution combustion method. The solution combustion method stands out as one of the best techniques due to its simplicity, rapid chemical process and its efficiency in saving both energy and time [23,24]. Additionally, this method produces highly homogeneous metal oxide NPs with a high surface area and a highly porous surface morphology [25,26]. It yields materials with excellent purity and very fine crystalline structures, offering a wide range of particle and crystalline sizes [27]. In the solution combustion method, the final physical and chemical properties of the NPs can be modified by controlling key experimental parameters, such as the nature of the fuel, metal precursors, pH, reaction temperature and the oxidizer-to-fuel stoichiometry. Some researchers have synthesized various metal oxide NPs via the solution combustion method using green and novel fuel such as plant extracts, flowers, plant latex and fruit juices. For example, Ahsani-Namin *et al*. successfully synthesized CuO/ZnO nanocomposites through green-mediated combustion using *Eryngium planum* leaf extract as a natural fuel. These nanocomposites were then applied to adsorb Congo red dye, with potential applications in the textile industry [28]. Naika *et al*. developed green solution synthesis of CuO NPs using *Gloriosa superba* extract and their antibacterial activity [29]. Madhukara Naik *et al*. synthesized green solution combustion synthesis of zinc doped cobalt ferrite NPs, and further applied this to photocatalytic and antibacterial applications [30]. Pavithra *et al*. synthesized ZnO NPs by the solution combustion method using citrus maxima (*Pomelo*) juice as green fuel and also evaluated their applications to photocatalytic, electrochemical sensor and antibacterial activities [31]. Osuntokun *et al*. synthesized ZnO NPs by the solution combustion method using *Brassica oleracea L. var. italica* as a fuel and studied its photocatalytic activity [32]. Some of the researchers showed more interest in the solution combustion synthesis of Ag/NiO NPs using plant based extract as a green fuel. For example, Malini *et al*. have developed green solution combustion synthesis of Ag–NiO NPs using *Acacia nilotica gum* as fuel and their evaluation and their synergetic effect on antimicrobial, anticancer and antioxidant activities [33]. Basavalingaiah *et al.* have synthesized NiO, and Ag–NiO NPs were prepared using *Lycopodium* extract as a green fuel via combustion method and applied to photocatalytic activity [34]. Hussain S *et al.* [35]*.* synthesized green synthesis of NiO NPs using *A. nilotica* leaf extracts and investigated their electrochemical and biological properties [35]. Ahamed *et al*. synthesized NiO NPs via the solution combustion method using *Aloe vera* gel extract as green fuel for application in supercapacitors [36].

This research focuses on an eco-friendly approach for synthesizing NiO and Ag/NiO NPs using *Cocos nucifera* (coconut) water both as a fuel and a solvent in the solution combustion method. The study also explores their applications in dye degradation, photocatalytic hydrogen production and antibacterial properties. The primary advantage of using *C. nucifera* water as a fuel in the solution combustion method is that it serves a dual purpose—both as a fuel and as a solvent. This eliminates the need for additional water to create a homogeneous redox mixture of fuel and oxidant in the experimental procedure. Moreover, *C. nucifera* water is non-toxic, eco-friendly and cost-effective. It is rich in sucrose, glucose and fructose, which are composed of carbon, hydrogen and oxygen. During combustion, these components are released as gases, resulting in the formation of highly porous NPs with a voluminous structure and a high surface area [25,26].

Organic dyes, such as trypan blue, are extensively used in the textile, food and paint industries for dyeing materials [28,29]. However, these dyes can cause significant environmental problems by releasing highly carcinogenic molecules into water bodies. Therefore, it is crucial to remove or degrade industrial effluents containing dyes to non-hazardous substances [27]. Given that dyes are toxic, carcinogenic and mutagenic, the World Health Organization has established a threshold limit of 0.9 mM for these substances in drinking water [29]. Consequently, monitoring their presence and implementing effective detoxification measures in various environmental matrices is of paramount importance [28,29]. Among the various methods available for monitoring dye contaminants in industrial wastewater, photocatalysis using NPs as photocatalysts and adsorbents is considered one of the most effective techniques for reducing the toxic content of dyes [30]. The current research article primarily focuses on the use of synthesized NiO and Ag/NiO NPs for the photocatalytic degradation of trypan blue dye. It thoroughly investigates various parameters that affect the rate of the photocatalytic degradation process, including pH, catalyst loading, dye concentration, light sources, light intensities, recyclability and the impact of scavengers.

Today, fossil fuels account for 50–60% of our energy needs [31]. However, fossil fuels are far from ideal due to their non-renewable nature, environmental impact, high costs and lower efficiency [32,33]. Therefore, it is essential to find an alternative energy source that is eco-friendly, cost-effective, renewable and more efficient. In this context, hydrogen (H_₂_) is considered a promising alternative to fossil fuels [34]. Among the various methods available for H_₂_ production, photocatalytic water splitting into H_₂_ and O_₂_ using synthesized NPs as photocatalysts is regarded as one of the best methods. Photocatalysis offers a straightforward approach to H_₂_ production from water, potentially converting solar energy into chemical energy in the form of H_₂_ [33,34]. The current research article primarily focuses on the use of synthesized NiO and Ag/NiO NPs for the photocatalytic water splitting reaction into H_₂_ production.

Over the past two to three decades, there have been significant advancements in the development of antibacterial drugs in the medical field [35,36]. Synthesized NPs have demonstrated enhanced antibacterial activity against pathogenic microbes, which pose serious threats to human health and the environment [36]. To combat these pathogens, antibiotics are commonly used. However, the overuse of antibiotics has led to the emergence of antibiotic-resistant bacterial strains, rendering many bacterial infections difficult to control with existing treatments [36,37]. As a result, developing antibacterial materials that do not rely on traditional antibiotics has become a major challenge. One promising approach to addressing this issue is to combine the photocatalytic activity of nanomaterials with the natural lifecycle of bacterial cells. Photocatalysis involves using light to drive chemical reactions, which can be harnessed to combat bacterial infections through novel mechanisms [38]. The current research article primarily focuses on the use of synthesized NiO and Ag/NiO NPs for the antibacterial activity against different pathogenic microbes.

## 2. Experimental procedures and methods

The starting materials and reagents used for the synthesis of NiO and Ag/NiO NPs were analytical-grade chemicals (approx. 99.9% purity), procured from Sigma-Aldrich, Bommasandra, Jigani Link Road, Industrial Area, Anekal Taluk, Bangalore 560010, Karnataka, India. These chemicals were used directly without further purification. For the production of NiO NPs, nickel nitrate was used as the nickel source, while for Ag/NiO NPs, silver nitrate served as the silver source. Fresh, naturally available *C. nucifera* water from India was used as fuel in the solution combustion method. A pictorial representation of the *C. nucifera* tree and its water is shown in electronic supplementary material, figure S1*a*,*b*.

### 2.1. Preparation of NiO and Ag/NiO NPs

For the production of NiO and Ag/NiO NPs, the following procedures were used.

#### 2.1.1. NiO NPs

Stoichiometric amounts of oxidizer (2.5 g of nickel nitrate) and fuel (35 mL of *C. nucifera* water) were mixed in a 250 ml borosilicate glass beaker. This redox mixture was stirred continuously for 15 min to ensure homogeneity. The homogeneous mixture was then pre-heated on a hot plate at 120°C for approximately 10 min, during which dehydration occurred and a gel formed. The gel was then subjected to combustion in a pre-heated muffle furnace at 450°C. The combustion process was smooth and smouldering, yielding a black nanocrystalline NiO powder within 5−6 min. The NiO NPs were further calcined at 600°C for 2 h to remove any impurities.

#### 2.1.2. Ag/NiO NPs

Various concentrations of Ag were doped into a NiO nanocrystalline structure. The doping was carried out with Ag contents of 0.1, 0.2, 0.3, 0.4 and 0.5 wt%. Stoichiometric amounts of oxidizing agents, such as nickel nitrate and silver nitrate, were combined with 35 ml of *C. nucifera* (coconut) water as the fuel. The mixture was prepared in a 250 ml borosilicate glass beaker, and this redox mixture was stirred continuously for 15 min to achieve homogeneity. The mixture was pre-heated on a hot plate at 120°C for about 10 min, leading to the formation of a gel. This gel was then subjected to combustion in a pre-heated muffle furnace at 450°C. The combustion process was smooth and smouldering, resulting in a black nanocrystalline Ag/NiO powder within 5−6 min. The Ag/NiO NPs were subsequently calcined at 600°C for 2 h to remove any impurities. The physical appearances of the synthesized NiO and Ag/NiO NPs are shown in electronic supplementary material, figure S1*c*,*d*, respectively. Flow chart and possible solution combustion reactions of NiO and Ag/NiO NPs using *C. nucifera* as fuel are given in the supplementary file as scheme 1.

### 2.2. Characterization

Powder X-ray diffraction (PXRD) of the prepared NiO and Ag/NiO NPs was analysed using a Rigaku II Cu-Kα system. PXRD analysis was conducted with Cu–Kα radiation (*λ* = 1.5418 Å), operating at 40 kV and 30 mA. The average crystallite size was determined from the peak with the highest intensity in the powder diffractogram, using Scherrer’s equation. Transmission electron microscopy (TEM) and high resolution transmission electron microscopy images (HRTEM) were obtained from the FEI Tecnai G2 S-model. The surface morphology and elemental distribution of the NiO and Ag/NiO NPs were analysed using scanning electron microscopy (SEM) and energy-dispersive X-ray spectroscopy (EDS), with data obtained from a Hitachi 3000 SEM. Fourier-transform infrared spectroscopy (FTIR) measurements were conducted using a SHIMADZU spectrophotometer. X-ray photoelectron spectroscopy (XPS) spectra of the synthesized NPs were recorded using a Thermo Scientific Al–Kα spectrometer as the excitation source. The UV spectral absorption and band gap of the synthesized NPs were investigated using a double-beam UV–visible (UV–vis) spectrophotometer (Schimadzu 2600). BET surface area and pore size of the synthesized NPs were analysed using an Micromeritics Tristar II surface area analyser with the N_2_ desorption–adsorption technique. A Horiba XploRA PLUS Raman microscope was used to record the Raman spectra.

### 2.3. Experimental procedure for photocatalytic dye degradation

A photocatalytic batch reactor (150 × 75 mm²), equipped with natural sunlight (965 W m^−^²) and UV light (175 W m^−^²), was used for photocatalytic dye degradation experiments. The experimental procedure involved adding a known amount of synthesized NiO and Ag/NiO NPs as photocatalysts to 100 ml of a 5 ppm dye solution. The mixture was continuously stirred in the dark for 30 min to achieve adsorption−desorption equilibrium. Following this, the mixture was irradiated with the light source. Every 15 min, an 8−10 ml sample of the dye slurry was collected, centrifuged at 1000 r.p.m. to remove the NiO and Ag/NiO NPs, and the solution’s absorbance was measured using a UV–vis spectrophotometer to determine the extent of dye degradation. The total percentage of dye degraded was calculated using the following equation (2.1) [15].


(2.1) $$\%\text{ of dye degradation }=\frac{A_{i}-A_{f}}{A_{i}}\times 100,$$

where *A_i_* is the initial absorbance of dye and *A_f_* is the absorbance of dye after 15 min of time duration.

### 2.4. Experimental procedure for H_2_ production via photocatalytic water splitting

The total amount of H_2_ gas that evolved from the photocatalytic water splitting reaction was monitored using a gas chromatograph (PerkinElmer ARNL 580C) equipped with a thermal conductivity detector, at room temperature in a water–ethanol system [18]. The experimental procedure involved dispersing approximately 10 mg of the synthesized NPs in 7.5 ml of water, followed by sonication for 15 min. Next, 2.5 ml of ethanol was added as a sacrificial reagent, and the mixture was illuminated with a light source. H_2_ production was measured at 30 min intervals using a gas-tight syringe with a maximum volume of 50 μl. The gas chromatograph equipped with a 5 Å molecular sieve-packed column and using argon as the carrier gas was employed for analysis [29].

### 2.5. Experimental procedure for antibacterial activity

The antibacterial activity of NiO and Ag/NiO NPs was tested against various bacterial strains, including Gram-positive bacteria *Staphylococcus aureus, Staphylococcus saprophyticus* and *Bacillus subtilis*, as well as Gram-negative bacteria *Pseudomonas aeruginosa*, *Escherichia coli, Klebsiella pneumoniae* and *Enterococcus faecalis*. The antibacterial efficacy of the NPs was evaluated using agar well diffusion and disc diffusion methods, with slight modifications from previous studies [34–36]. Pure broth cultures of each bacterial strain were incubated for 24 h and grown on nutrient agar plates using the spread plate method with a sterilized L-shaped glass rod. The 6 mm wells were then created on each plate using a sterile cork borer. Solutions of the NPs at concentrations of 200, 100, 50 and 25 mg per well were prepared by dispersing the NPs in double-distilled water and were added to wells 1, 2, 3 and 4, respectively. The NPs were serially diluted from the stock solution of the first well. Fifty microliters of each NP dilution were added to the corresponding wells. *Penicillin* (30 µg ml^−1^; Hi-Media) was used as the positive control and placed in the centre of the plate, while double-distilled water served as the negative control in the fifth well. The plates were incubated in a biological oxygen demand incubator at 37°C for 24–36 h. Antibacterial activity was assessed by measuring the diameter of the clear zones of inhibition around the bacterial growth [30].

## 3. Results and discussion

### 3.1. XRD studies

**Figure 1.**
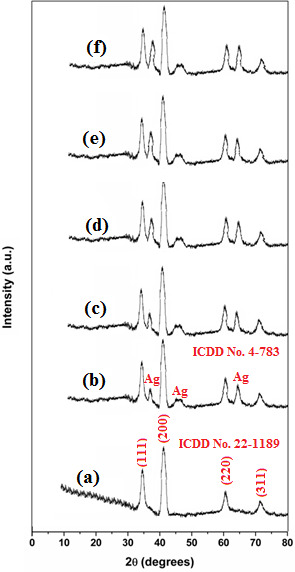
PXRD patterns of (*a*) undoped NiO NPs, (*b*) 0.1 wt% Ag/NiO NPs, (*c*) 0.2 wt% Ag/NiO NPs, (*d*) 0.3 wt% Ag/NiO NP, (*e*) 0.4 wt% Ag/NiO NPs and (*f*) 0.5 wt% Ag/NiO NPs.


The phase purity and crystalline structure of the synthesized NPs were investigated using the XRD technique. Figure 1(a) presents the XRD pattern of undoped NiO NPs synthesized via the solution combustion method. The XRD data were recorded over a 2θ range from 0° to 80°. Diffraction peaks observed at 37.2°, 42.1°, 63° and 74.5° correspond to the (111), (200), (220) and (311) planes, respectively (*International Centre for Diffraction Data (ICDD) number: 22-1189*), and are indexed to a cubic phase with a rhombohedral structure. The lattice parameters are *a* = *b* = 3.214 Å and *c* = 4.532 Å. The average crystallite size of the undoped NiO NPs, determined using Scherrer’s equation, i.e. equation (3.1), was found to be 31.5 nm. The absence of any additional peaks, apart from those corresponding to NiO NPs, indicates the high phase purity of the synthesized NPs. Figure 1(b)–(f) illustrates the XRD patterns for Ag-doped NiO NPs with Ag concentrations ranging from 0.1 to 0.5 wt%. When comparing these patterns with those of undoped NiO NPs, we observed not only the diffraction peaks of NiO but also three additional peaks at 38.3°, 44.1° and 64.6° *(ICDD number: 4-783)* corresponding to the cubic phase of Ag. This indicates that Ag was successfully incorporated into the NiO nanocrystalline structure. As the concentration of Ag increased from 0.1 to 0.5 wt%, the peak intensities associated with Ag also increased. The average crystallite sizes of the Ag/NiO NPs, determined using equation (3.1), were found to be 35.5 nm for 0.1 wt% Ag, 37.8 nm for 0.2 wt% Ag, 39.5 nm for 0.3 wt% Ag, 42.5 nm for 0.4 wt% Ag and 44 nm for 0.5 wt% Ag. This trend shows an increase in average crystallite size with increasing Ag concentration. This growth can be attributed to the higher Ag content weakening the internal structure of the NiO nanostructure, which accelerates nucleation and particle growth [14].

(3.1) $$D=\frac{K\lambda }{\beta \cos\theta },$$

where *D* is the average crystallite size, *λ* is the X-ray wavelength in nm, *β* is the full-width at half-maximum resulting from small crystallite size in radians and *K* is a constant related to crystallite shape, normally taken as 0.9 and *θ*: the Bragg angle.

### 3.2. FTIR studies

**Figure 2.**
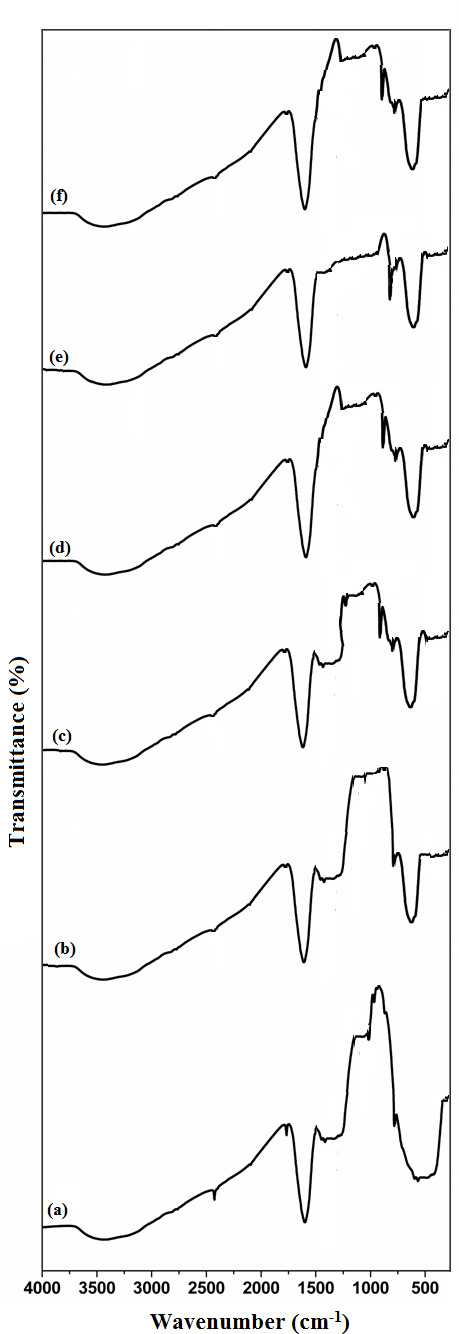
FTIR spectra of (*a*) undoped NiO NPs, (*b*) 0.1 wt% Ag/NiO NPs, (*c*) 0.2 wt% Ag/NiO NPs, (*d*) 0.3 wt% Ag/NiO NP, (*e*) 0.4 wt% Ag/NiO NPs and (*f*) 0.5 wt% Ag/NiO NPs.

FTIR studies revealed the presence of various functional groups in the synthesized NPs. Figure 2(a) displays the FTIR spectrum of undoped NiO NPs, while figure 2(b)–(f) shows the spectra for Ag/NiO NPs with concentrations ranging from 0.1 to 0.5 wt%. From the FTIR studies, the absorption band observed at the range 460−900 cm^−1^ may be attributed to stretching vibrations of the metal–oxygen bands (Ni–O and Ag–O bands) [8–10]. Broad FTIR peak at around 3454 cm^−1^, which related to the stretching vibrations of O–H molecules in H_2_O [13] and FTIR peak at range 1648 cm^−1^ is assigned to the bending vibrations of H–O–H molecules in H_2_O [23]. Overall, the FTIR studies indicate that there are no additional characteristic peaks related to organic contaminants in the synthesized NPs. This suggests a high level of purity in the NPs synthesized via the solution combustion method using *C. nucifera* water as the fuel. The FTIR spectrum of *C. nucifera* water is presented in the electronic supplementary material, figure S2. *Cocos nucifera* contains various nutrients, including fructose, glucose, sucrose, amino acids and electrolytes such as potassium, calcium and magnesium, as well as proteins and vitamins. The FTIR bands at 629 and 2799 cm^−1^ correspond to the symmetric C–H stretching of =CH_2_ (alkenes), and the asymmetric C–H stretching of =CH_2_ appears at 2921 cm^−1^ [39]. The band at 1067 cm^−1^ indicates the presence of the C–O group in esters or carboxylic acids [40]. A band at 1410 cm^−1^ is associated with the C=C bond of an allyl group in alkenes [9]. An N–H band is observed around 1640 cm^−1^ [40], and the O–H band at 3421 cm^−1^ [39] is related to the hydroxyl group of -COOH or adsorbed structural water. Based on the FTIR analysis results and the reducing properties of N–H and C–H related functional groups, *C. nucifera* can be used as a fuel in redox reactions involved in solution combustion synthesis.

### 3.3. UV–Vis, band gap studies and determination of quantum efficiency

**Figure 3.**
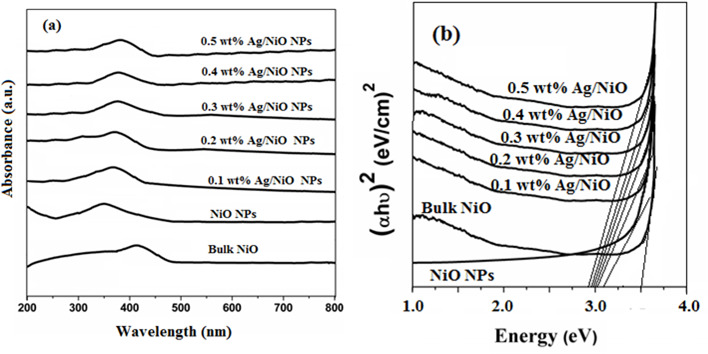
(*a*) UV–Vis spectra of bulk NiO, NiO and 0.1–0.5 wt% Ag/NiO NPs and (*b*) Tauc plots of bulk NiO, NiO and 0.1–0.5 wt% Ag/NiO NPs.

The optical properties of the synthesized NPs were investigated using UV–Vis spectroscopy, with spectra recorded in the range of 200−800 nm. Figure 3(a) displays the UV–Vis spectrum of undoped NiO NPs, which shows a maximum absorption peak at 345 nm. This corresponds to a band gap value of 3.21 eV. This value is blue-shifted compared to bulk NiO particles (maximum absorption peak at around 410 nm), due to quantum confinement effects [12,13]. Figure 3(a) presents the UV–Vis spectra for Ag/NiO NPs with concentrations ranging from 0.1 to 0.5 wt%. After doping Ag into the NiO nanostructure, the maximum absorption peak shifted to longer wavelengths in the case of 0.1–0.5 wt% Ag-doped NiO NPs, around 365−369 nm with a band gap of 3.03−2.87 eV, indicating a red shift compared to the undoped NiO NPs [16,17]. This red shift confirms the successful incorporation of Ag into the NiO structure. Additionally, the Ag/NiO NPs demonstrated stronger absorption compared to the undoped NiO NPs [19]. This enhanced absorption is associated with improved photocatalytic and antibacterial activities in the Ag/NiO NPs compared to their undoped NiO NPs [24,25]. The band gap energy of the synthesized NPs was determined using Tauc plots, with the corresponding graphs shown in figure 3(b). Quantum efficiency of the synthesized NPs was determined using a double-beam UV–Vis spectrometer and spectrofluorometer using flowing equation (3.2) [38].


(3.2) $$Q_{NPs}=\frac{Q_{\text{standard }}X\;\left(Slope\right)\;NPs\;X\;\eta^{2}NPs}{{\left(Slope\right)}_{NPs}\;X\;\eta_{\text{ standard }}^{2}}$$

Here, *Q* is the quantum efficiency and *η* is the refractive index of the solvent used in the experiment. Slope is calculated by graph taking absorbance against fluorescence intensity. The quantum efficiency values were as follows: undoped NiO NPs had a quantum efficiency of 0.534, while the 0.1 wt% Ag/NiO NPs had a quantum efficiency of 0.623, the 0.2 wt% Ag/NiO NPs had 0.679, the 0.3 wt% Ag/NiO NPs had 0.781, the 0.4 wt% Ag/NiO NPs had 0.692 and the 0.5 wt% Ag/NiO NPs had 0.683. The quantum efficiency of the Ag/NiO NPs was higher than that of the undoped NiO NPs. This enhancement is likely due to the synergistic effect of Ag, which acts as an electron sink by accepting electrons from the conduction band of NiO [40]. This process reduces electron–hole recombination and improves the photocatalytic activity of the Ag/NiO NPs compared to the undoped NiO NPs [41]. When further comparing the quantum efficiencies of different Ag-doped NiO NPs, it was proved that among five different Ag concentrations, 0.3 wt% Ag/NiO NPs showed higher quantum efficiency than the rest and the synergy between the Ag and NiO has an optimum for concentration of 0.3 wt% Ag-doped NiO. Based on UV–Vis spectroscopy, band gap measurements and quantum efficiency studies, it was observed that the 0.3 wt% Ag-doped NiO NPs exhibited superior photocatalytic and antibacterial activities compared to the other synthesized NPs.

### 3.4. Photoluminescence (PL) studies

**Figure 4.**
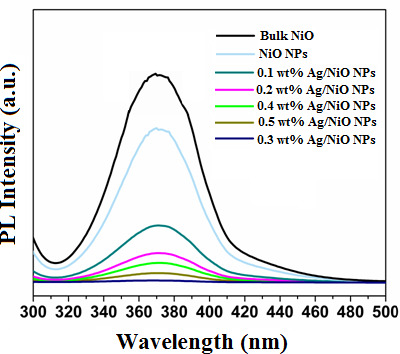
PL spectra of bulk NiO particles, NiO NPs and 0.1–0.5 wt% Ag/NiO NPs.

To investigate the impact of Ag doping on the NiO nanocrystalline structure and to understand the separation and movement of charge carriers in the synthesized NPs, PL spectroscopy was performed. PL spectroscopy was conducted by exciting both bulk NiO particles, undoped NiO and Ag/NiO NPs with a 350 nm excitation wavelength, with the results shown in figure 4. PL spectra of bulk NiO particles, undoped NiO NPs and Ag/NiO NPs were similar in terms of emission wavelength, with a prominent peak around 372 nm. However, the relative intensities of these peaks varied among the samples. Bulk NiO particles exhibited a higher PL intensity than the NPs, indicating more rapid recombination of excited electrons from the conduction band with holes in the valence band. In contrast, undoped NiO NPs had a higher PL intensity compared to Ag/NiO NPs, suggesting that the recombination of excited electrons and holes was more rapid in the undoped NPs. This enhanced recombination in bulk NiO particles and undoped NiO NPs likely contributed to their lower photocatalytic activity compared to Ag/NiO NPs. The reduced PL intensity in Ag/NiO NPs implies that Ag doping decreases the recombination rate of charge carriers, thereby improving the photocatalytic and surface properties of the NPs. Further analysis of PL intensities with varying Ag concentrations revealed that 0.3 wt% Ag/NiO NPs had the lowest emission intensity among the samples. This suggests that 0.3 wt% Ag doping is optimal for reducing the recombination rate of photoexcited electrons and holes. At lower Ag concentrations (0.1 and 0.2 wt %), there are not enough trap states to significantly affect recombination rates. Conversely, higher Ag concentrations (0.4 and 0.5 wt %) can hinder light absorption and electron–hole generation. Therefore, PL spectroscopy indicates that a 0.3 wt% Ag doping concentration effectively introduces donor levels, defect levels and surface trap states within the NiO nanocrystalline structure, which enhances charge carrier separation and reduces recombination.

## 4. Photocatalytic activity of NiO and Ag/NiO NPs

### 4.1. Photocatalytic water splitting reaction into H_2_ generation

**Figure 5.**
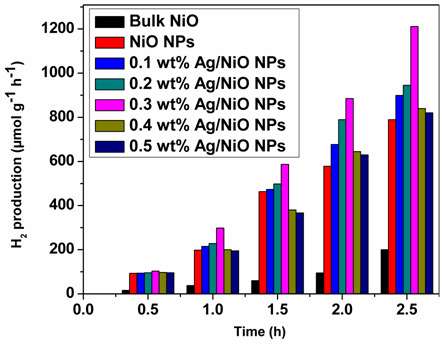
Photocatalytic H_2_ production using various photocatalysts.

To determine which synthesized NPs are most effective for photocatalytic water splitting and H_₂_ generation, the performance of various NPs was evaluated in terms of their H_₂_ production capabilities and shown in figure 5. Experimental results demonstrated that all the synthesized NPs exhibited substantial H_₂_ generation, with production increasing linearly over time. NiO and Ag/NiO NPs exhibited higher photocatalytic H_₂_ production compared to bulk NiO particles. These results highlight the superior photocatalytic activity of nanosized materials over their bulk counterparts. Furthermore, when comparing the photocatalytic activities of NiO and Ag/NiO NPs, the Ag-doped NiO NPs demonstrated superior H_₂_ production compared to the NiO NPs. These results underscore the significance of doping noble metals into transition metal oxide nanostructures for enhanced photocatalytic performance. The photocatalytic H_₂_ production of NiO nanostructures with varying Ag concentrations revealed that among five different Ag concentrations, 0.3 wt% Ag-doped NiO NPs exhibited the highest H_₂_ production (1212 µmol g⁻¹ h^-1^) compared to the other samples. These results indicate that 0.3 wt% Ag/NiO NPs are the most effective photocatalysts for H_₂_ production. A pictorial representation of the movement of electrons and holes in 0.3 wt% Ag/NiO NPs for photocatalytic H_₂_ production is shown in electronic supplementary material, figure S3.

### 4.2. Photocatalytic dye degradation studies

**Figure 6.**
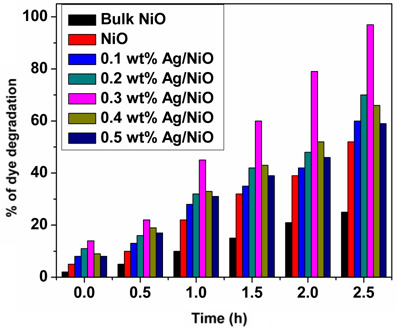
Photocatalytic dye degradation using various photocatalysts.

To select the optimal photocatalyst from various synthesized nanomaterials produced using the solution combustion method for trypan blue dye degradation, each material was tested for its photocatalytic performance. The results of these dye degradation tests are presented in figure 6. Similar to the results observed in photocatalytic H_2_ production, the synthesized NPs demonstrated a significantly higher rate of dye degradation compared to bulk NiO particles. This finding underscores the advantage of nanosized materials over bulk particles. The enhanced photocatalytic activity of the NPs can be attributed to their larger surface area-to-volume ratio and the quantum confinement effects inherent in nanoscale materials. Next, it was observed that Ag-doped NiO NPs exhibited a higher rate of dye degradation compared to undoped NiO NPs. This result highlights the significant role of the doping agent (Ag) in reducing the recombination rate of photoexcited electron–hole pairs, thereby enhancing the photocatalytic performance beyond that of the undoped NiO NPs. Among the five different Ag concentrations in NiO nanostructures, the 0.3 wt% Ag-doped NiO NPs demonstrated the highest dye degradation efficiency, achieving 98% degradation within 2.5 h. These results indicate that the 0.3 wt% Ag/NiO NPs is the most effective photocatalyst for dye degradation among the tested samples. A pictorial representation of the movement of charge carriers (electrons and holes) in 0.3 wt% Ag/NiO NPs during photocatalytic dye degradation is illustrated in electronic supplementary material, figure S4. A comparison table of photocatalytic dye degradation performance by various metal oxide and metal-doped metal oxide NPs is presented in the electronic supplementary material, table S1, along with the current study.

The photocatalytic H_₂_ production and dye degradation studies demonstrated that 0.3 wt% Ag/NiO NPs exhibited superior photocatalytic activity compared to the other tested samples. This enhanced performance can be attributed to several factors, including higher quantum efficiency, a larger surface area, precisely tuneable band gap values and a more porous and voluminous surface morphology. In this research article, the 0.3 wt% Ag/NiO NPs were further characterized in detail to understand their physical and chemical properties.

## 5. Additional characterization studies of 0.3 wt% Ag/NiO NPs

### 5.1. XPS analysis

**Figure 7.**
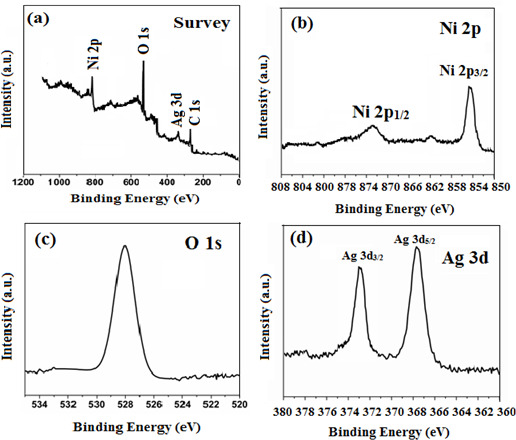
XPS spectra of (*a*) survey spectrum of 0.3 wt% Ag/NiO NPs, (*b*) Ni 2p, (*c*) O 1s and (*d*) Ag 3d.

The chemical composition and oxidation states of 0.3 wt% Ag/NiO NPs were analysed using XPS [42]. The broad XPS survey spectrum of 0.3 wt% Ag/NiO NPs (figure 7(a)) identified the presence of Ni, O and Ag, confirming both the successful formation of the NiO nanostructure and the effective doping of Ag into the NiO matrix. These findings are in good agreement with previous studies [5,6]. Figure 7(b) shows the XPS spectrum of the Ni 2p core level, with binding energies at 854.5 eV for Ni 2 _p3/2_ and 872.5 eV for Ni 2 _p1/2_ [17,18]. Figure 7(c) displays the XPS spectrum of the O 1s core level, with a binding energy of 529.2 eV, which corresponds to lattice oxygen in Ag/NiO [27]. Figure 7(d) presents the XPS spectrum of the Ag 3d core level, showing two characteristic peaks with binding energies of 373.8 eV for Ag 3d_3/2_ and 367.9 eV for Ag 3d_5/2_. The observed binding energy difference of 5.9 eV between Ag 3d_3/2_ and Ag 3d_5/2_ indicates the presence of metallic Ag, consistent with previous studies [28,29].

### 5.2. SEM and EDS studies

The surface morphology, including the shape, size and distribution of NPs in the synthesized 0.3 wt% Ag/NiO NPs, was examined using SEM. Electronic supplementary material, figure S5*a* presents the SEM image of the 0.3 wt% Ag/NiO NPs presented in the supplementary material, which were synthesized via the solution combustion method using *C. nucifera* water as fuel. The image reveals a voluminous, porous surface morphology with numerous voids. This porous structure results from the release of various combustible gases during the complete combustion process. The voluminous, porous morphology with abundant voids contributes to the enhanced photocatalytic and antibacterial activities of the 0.3 wt% Ag/NiO NPs [12]. The chemical composition and quantitative atomic percentages of the constituents in 0.3 wt% Ag/NiO NPs were analysed using EDS. Electronic supplementary material, figure S5*b* shows the EDS results for the 0.3 wt% Ag/NiO NPs, revealing the presence of Ag, Ni and O elements. The atomic percentage ratios were found to be 48.2% for Ni, 44.5% for O and 7.3% for Ag. The EDS analysis confirms the presence of Ag in the NiO nanostructure and indicates that the Ag particles are uniformly distributed throughout the Ag/NiO sample.

### 5.3. TEM studies

**Figure 8.**
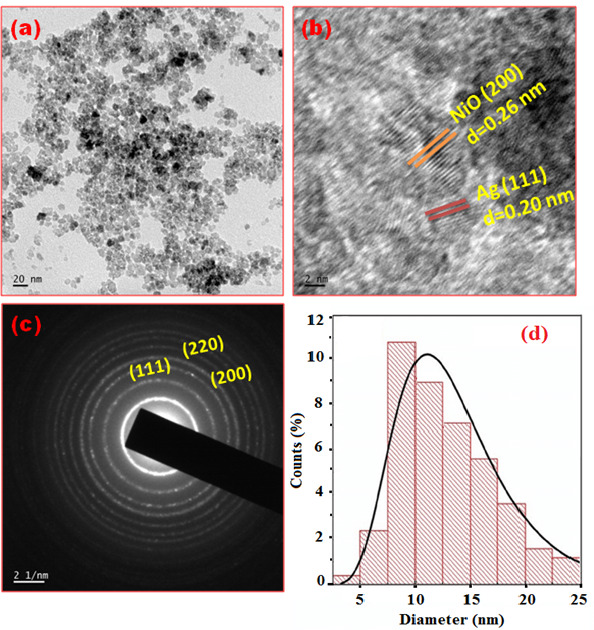
(*a*) TEM image, (*b*) HRTEM image (*c*) SAED pattern and (*d*) particle size distribution histogram of 0.3 wt% Ag/NiO NPs.

The crystal size, growth, shape and distribution of the synthesized 0.3 wt% Ag/NiO NPs were examined using TEM. Figure 8(a) presents the TEM image of the 0.3 wt% Ag/NiO NPs, revealing that most particles are spherical, irregular in size, and well-distributed with minimal aggregation that occurred during synthesis with average particle size of 25.8 nm. Figure 8(b) shows an HRTEM image of the 0.3 wt% Ag/NiO NPs, with lattice spacings of 0.26 and 0.20 nm, corresponding to the (200) and (111) planes of NiO and Ag phases, respectively. Figure 8(c) displays the SAED pattern of the 0.3 wt% Ag/NiO NPs, confirming their polycrystalline nature with reflections from the (200) and (220) planes of NiO and the (111) plane of Ag, consistent with the XRD studies. The particle size was estimated using ImageJ software, analysing nearly 100 particles from various TEM images to create the histogram shown in figure 8(d). The corresponding histogram is fitted with a log-normal distribution curve (black line), which yields a mean size of 10.5 ± 0.54 nm. These values are in good agreement with the XRD data.

### 5.4. Raman studies

The vibration modes and structural defects in the synthesized NiO and 0.3 wt% Ag/NiO NPs were studied using Raman spectroscopy. Electronic supplementary material, figure S6, displays the room temperature Raman spectra of these NPs. The Raman spectrum of 0.3 wt% Ag/NiO NPs reveals the following four characteristic peaks: the first peak at approximately 517 cm⁻¹ corresponds to the first-order longitudinal-optical (1LO) mode, which is associated with Ni–O vibrations [43]; the second peak at approximately 1070 cm⁻¹ is attributed to the second-order longitudinal-optical (2LO) mode [44]; the third peak at approximately 703 cm⁻¹ is due to the second-order transverse-optical (2TO) phonon mode [44,45]; and the fourth peak at approximately 908 cm⁻¹ results from the combination of longitudinal-optical and transverse-optical phonon modes (TO + LO) [43]. Compared to undoped NiO NPs, the 0.3 wt% Ag/NiO NPs exhibit a slight red shift in these peaks. For undoped NiO NPs, the Raman spectra show characteristic peaks at 520 (1LO), 1078 (2LO), 703 (2TO) and 910 cm⁻¹ (TO + LO) [45]. The observed red shifts and the absence of additional peaks confirm the successful doping of Ag into the NiO nanostructure, as well as the high purity and cubic phase of the synthesized NPs, which is consistent with the XRD results.

### 5.5. BET surface area analysis

**Figure 9.**
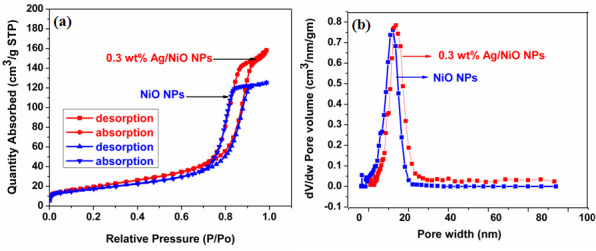
(*a*) BET measurements of NiO and 0.3 wt% Ag/NiO NPs. (*b*) Pore size distributions of NiO and 0.3 wt% Ag/NiO NPs.

The surface properties, including surface area and porous structure, of the synthesized NiO and 0.3 wt% Ag/NiO NPs were analysed using a BET surface area analyser. Figure 9(a) presents the BET curves for both NiO and 0.3 wt% Ag/NiO NPs. Both samples exhibited type IV adsorption isotherms, indicating key surface characteristics such as a mesoporous structure and a clear monolayer–multilayer adsorption process [13]. Previous studies have demonstrated that NPs with type IV adsorption isotherms possess a significant catalytic advantage: they facilitate the efficient separation of adsorbates from the absorbent during the photocatalysis process [21–23]. It was observed that the 0.3 wt% Ag/NiO NPs exhibited a greater surface area (170 m^2^ g^−1^) compared to the NiO (130 m^2^ g^−1^) NPs. This increased surface area suggests that the 0.3 wt% Ag/NiO NPs are more effective at adsorbing surrounding molecules than the NiO NPs alone, which contributes to their enhanced photocatalytic activity [38]. The pore diameters of the NiO (15.1 nm) and 0.3 wt% Ag/NiO NPs (17.5 nm) are shown in figure 9(b).

## 6. Experimental factors affecting photocatalytic the rate H_2_ production

Various factors, including light sources, illumination intensities, pH levels and the reusability of the photocatalyst, influence the rate of photocatalytic H_2_ production [13–15]. To evaluate the effect of different light sources on this rate, photocatalytic experiments were performed under the following three conditions: UV light, visible light and in the absence of light (dark medium). The results, shown in electronic supplementary material, figure S7*a*, indicate that 0.3 wt% Ag/NiO NPs exhibited a higher rate of photocatalytic H_2_ production under light exposure compared to no light, with almost no H_2_ production observed in the dark. This highlights the vital role of light in facilitating effective charge carrier separation for photocatalytic H_2_ production. Furthermore, 0.3 wt% Ag/NiO NPs demonstrated a higher rate of H_2_ production under visible light (1200 µmol g⁻¹ h⁻¹) compared to UV light (958 µmol g⁻¹ h⁻¹). The enhanced photocatalytic H_2_ production under visible light is attributed to the lower band gap, higher quantum yield, larger surface area and the voluminous, porous surface morphology of the 0.3 wt% Ag/NiO NPs. Illumination intensity significantly impacts the rate of photocatalytic H_2_ production [37]. To investigate this, photocatalytic experiments were conducted in a dark medium and under Xe arc light sources with intensities of 200 and 400 W. The amount of H_2_ produced was measured and is shown in electronic supplementary material, figure S7*b*. In the dark medium, no H_2_ was produced. However, with increasing light intensity, the rate of H_2_ production also increased. Specifically, the H_2_ production rates were 1005 and 1220 µmol g⁻¹ h⁻¹ under 200 and 400 W Xe arc light intensities, respectively. These results demonstrate that higher light intensities increase the number of incident photons, which in turn promotes more electrons into the conduction band of the NiO semiconducting metal oxides. These electrons are then accepted by noble metal dopants like Ag, leading to enhanced separation of electrons and holes and thereby increasing the rate of photocatalytic H_2_ production. Surface charge, surface agglomeration, adsorption behaviour and the band gap value of the synthesized 0.3 wt% Ag/NiO NPs are significantly influenced by pH values [33–35]. Although understanding the precise effects of medium pH on photocatalytic H_2_ production can be complex, previous research has indicated that an acidic medium with a pH range of 2−6 is more favourable for photocatalytic H_2_ production compared to a basic medium with a pH range of 7.5−12 [28,29]. To further explore the effect of pH on photocatalytic H_2_ production, the pH of the medium was varied from 2 to 10, and photocatalytic experiments were conducted under UV light illumination. The results, shown in electronic supplementary material, figure S7*c*, indicate that the maximum H_2_ production (999 µmol g⁻¹ h⁻¹) occurred at pH 4, compared to 645 µmol g⁻¹ h⁻¹ at pH 8−10. This enhancement in H_2_ production at pH 4 can be attributed to the increased adsorption of H^+^ ions on the surface of the 0.3 wt% Ag/NiO photocatalyst, which facilitates a higher rate of reduction of H^+^ ions to H_2_. In the present study, the 0.3 wt% Ag/NiO photocatalyst exhibited the highest hydrogen production at pH 4. However, at a lower pH of 2 (more acidic), hydrogen production decreased. This reduction may be attributed to increased particle agglomeration at pH 2 compared to pH 4. The phenomenon was strongly influenced by the pH value, as the acidity or alkalinity of the solution can affect the electrostatic forces between particles, leading to either increased or decreased particle clumping. Specifically, a lower pH (more acidic) often results in greater agglomeration due to reduced electrostatic repulsion between particles. Increased agglomeration reduces the available surface area for the reaction, thus significantly limiting hydrogen production [20–23]. At higher pH values (more basic), in a basic environment with a high pH, there are fewer protons available, making it less likely for a molecule to accept a proton and become protonated; resulting hydrogen production decreased at higher pH in the present work. One of the key features for an ideal photocatalyst was recyclability/durability. To evaluate the recyclability and durability of the 0.3 wt% Ag/NiO photocatalyst, photocatalytic experiments were conducted over six repeated cycles. The results, shown in electronic supplementary material, figure S7*d*, demonstrate an average H_₂_ production across all six cycles, indicating that the photocatalyst exhibits moderate photostability. However, a significant decrease in the H_₂_ evolution rate was observed after the third cycle. This decline can be attributed to the loss of photocatalyst mass. After each cycle, the mass of the recycled 0.3 wt% Ag/NiO photocatalyst was measured and found to decrease from 10 to 8.5 mg by the third cycle. This reduction in mass likely contributed to the lower H_₂_ production observed in subsequent cycles. Another contributing factor to the decreased H_₂_ production could be the treatment of the NPs after the first cycle. The NPs were extracted, washed twice and dried at 80°C in a hot-air oven for 2 h before being reused. The photocatalytic activity of the recycled catalyst showed a significant reduction, most likely due to aggregation and sedimentation of the NPs. This led to parts of the catalyst surface becoming unavailable for dye adsorption and photon absorption, which in turn reduced the efficiency of the catalytic reaction. Overall, the study confirms that the 0.3 wt% Ag/NiO photocatalyst maintains average photocatalytic activity and can be reused, although its performance diminishes with repeated cycles.

## 7. Experimental factors affecting photocatalytic dye degradation

**Figure 10.**
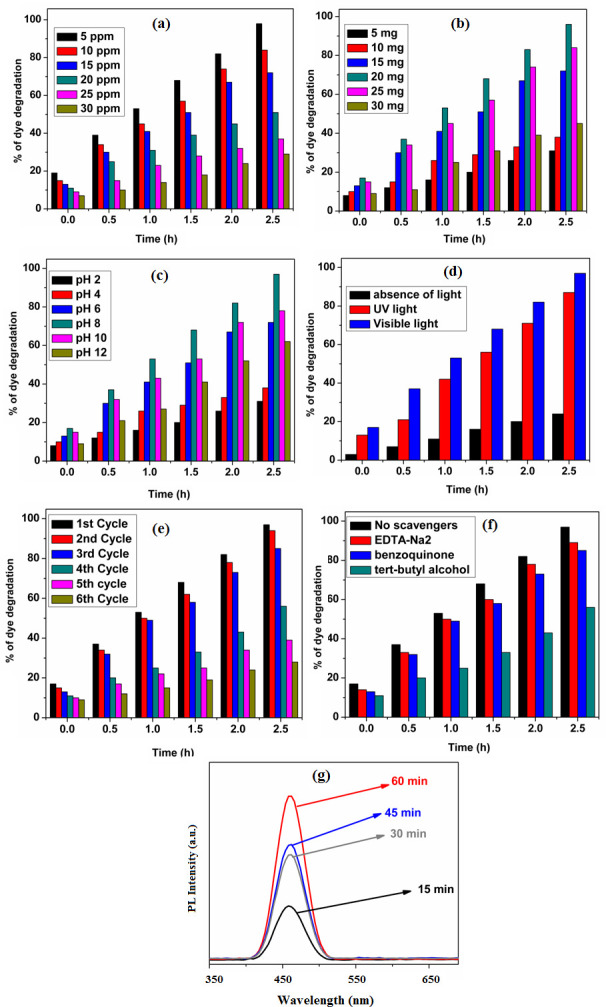
(*a*) Effect of concentration of dye, (*b*) effect of catalytic load, (*c*) effect of pH, (*d*) effect of different lights, (*e*) effect of recyclability, (*f*) scavenger studies and (*g*) detection of OH radicals of 0.3 wt% Ag/NiO photocatalyst on dye degradation.

To assess the photocatalytic efficiency of 0.3 wt% Ag/NiO NPs in dye degradation, we investigated the effects of various factors. These included dye concentration, catalyst loading, pH, different light sources and the recyclability of the photocatalyst [14–16]. Figure 10(a) illustrates the impact of dye concentration on the photocatalytic activity of 0.3 wt% Ag/NiO NPs. The experiment was conducted by varying the dye concentrations from 5 to 30 ppm at pH 8, using a catalyst amount of 20 mg and UV light. A dye concentration of 5 ppm exhibited the highest photocatalytic degradation efficiency. At higher dye concentrations, UV light penetration becomes more challenging, reducing its ability to reach the catalyst surface. This limitation impedes the generation of OH radicals, which are crucial for dye degradation. Additionally, at elevated concentrations, the dye molecules absorb UV light more effectively than the photocatalyst, further diminishing OH radical production and thus decreasing dye degradation. From these observations, it can be concluded that for effective dye degradation, there must be a balance between the catalytic load and dye concentration [7–10]. To assess the impact of catalytic load on the rate of dye degradation, an experiment was conducted by varying the catalyst amount from 5 to 30 mg while maintaining a dye concentration of 5 ppm. The results are shown in figure 10(b). Dye degradation increased linearly with the catalytic load from 5 to 20 mg. However, at higher catalytic loads (25 and 30 mg), dye degradation decreased due to increased agglomeration and sedimentation of the photocatalyst. This clustering reduces the available surface area and active sites on the catalyst, impeding the degradation process. These findings indicate that 20 mg is the optimal catalytic load for effective dye degradation in this study. Figure 10(c) illustrates the effect of pH on the dye degradation process. The photocatalytic experiment was conducted by varying the pH from 2 to 12, while keeping the dye concentration at 5 ppm and the catalytic amount at 20 mg. At lower pH values (2–6), dye degradation efficiency decreased because the dye molecules strongly adsorbed onto the photocatalyst surface. This adsorption reduced the number of available active sites and minimized the generation of OH radicals. At higher pH values (9–12), dye degradation also decreased due to changes in the surface charge of the catalyst [23–25]. According to the zero point of charge of 0.3 wt% Ag/NiO NPs, which is approximately 8 ± 0.1, the catalyst surface becomes negatively charged at pH values above 8 due to increased adsorption of OH⁻ ions. This results in fewer OH radicals being generated on the catalyst surface, thereby reducing dye degradation. The optimum pH for effective dye degradation was found to be around 8 in this study. To evaluate the impact of different light sources on dye degradation, photocatalytic experiments were conducted under the following three conditions: in the absence of light, with UV light and with visible light. The experimental results are presented in figure 10(d). In the absence of any light source, the degradation efficiency of the catalyst decreased, highlighting the crucial role of light in the dye degradation process. Furthermore, the 0.3 wt% Ag/NiO NPs demonstrated higher degradation efficiency under visible light compared to UV light. This improved performance under visible light is attributed to the lower band gap, higher quantum yield, larger surface area and the porous surface morphology of the 0.3 wt% Ag/NiO NPs. Figure 10(e) illustrates the reusability of the 0.3 wt% Ag/NiO NPs for dye degradation. The experiment was conducted with a catalyst load of 20 mg, at pH 8, and a dye concentration of 5 ppm. The catalyst did not maintain the same degradation efficiency after six cycles. A significant decrease in efficiency was observed after the fourth cycle. This decline is attributed to the loss of catalyst during repeated usage and filtration, which negatively impacted dye degradation performance in the fifth and sixth cycles. The photocatalytic activity of the recycled catalyst showed a notable reduction, likely due to aggregation and sedimentation of the dye on the catalyst surface. As a result, parts of the catalyst surface became unavailable for dye adsorption and photon absorption, leading to a decrease in catalytic efficiency. Overall, the study confirms that the 0.3 wt% Ag/NiO photocatalyst retains moderate photocatalytic activity and can be reused, though its performance diminishes with repeated cycles. In general, holes (h^+^), hydroxyl radicals (^•^OH) and superoxide anion radicals (O_2_^•-^) are considered the most important reactive species for dye degradation [33–35]. In the present study, scavenger trapping experiments were conducted to evaluate the roles of these reactive species for dye degradation. The scavenging agents used included the disodium salt of ethylene diamine tetra acetic acid (EDTA-Na_2_, 2 mM) for holes, benzoquinone (2 mM) for superoxide anion radicals and tert-butyl alcohol (2 mM) for hydroxyl radicals. The corresponding results are presented in figure 10(f). After adding all three scavenging agents during the dye degradation experiments, a significant decrease in dye degradation was observed. This indicates that holes, superoxide anion radicals and hydroxyl radicals play an important role in dye degradation in the present study. The scavenger trapping experiments demonstrated that hydroxyl radicals are among the most important reactive species for dye degradation. These hydroxyl radicals, generated during the degradation process, are highly unstable and reactive. To detect hydroxyl radicals in this study, a PL instrument was used with coumarin as a probe molecule [46]. The experimental results are presented in figure 10(g). In this experiment, the coumarin molecule reacts with hydroxyl radicals to form the fluorescent compound 7-hydroxycoumarin, which exhibits an excitation peak at 455 nm in PL spectroscopy. To conduct the experiment, 20 mg of 0.3 wt% Ag/NiO NPs was mixed with 80 ml of a 10^−3^ M coumarin solution. To achieve adsorption–desorption equilibrium, the coumarin solution was allowed to interact with the 0.3 wt% Ag/NiO NPs photocatalyst before and after irradiation with light source. Every 15 min, 5 ml of the solution was collected, and the generated hydroxyl radicals were evaluated using a PL instrument. The peak intensity at 455 nm gradually increased with irradiation time, indicating a rise in hydroxyl radicals, which correspondingly enhanced the overall concentration of 7-hydroxycoumarin molecules.

## 8. Antibacterial property of the 0.3 wt% Ag/NiO NPs

The diffusion assay was employed to investigate the antibacterial properties of 0.3 wt% Ag/NiO NPs at various concentrations against the following three Gram-positive bacteria: *S. aureus, S. saprophyticus* and *B. subtilis*, as well as the following four Gram-negative bacteria: *P. aeruginosa*, *E. coli, K. pneumoniae* and *E. faecalis*. The results are presented in electronic supplementary material table S2, and it illustrates the inhibitory zone values for various bacterial strains. The results show that among the tested pathogens, the Gram-negative bacterium *E. coli* exhibited the largest inhibitory zone as the concentration of 0.3 wt% Ag/NiO NPs increased. The other pathogens also displayed significant zones of inhibition with rising concentrations of Ag/NiO NPs, as shown in the electronic supplementary material, figure S8. The antibacterial properties of the 0.3 wt% Ag/NiO NPs can be attributed to their ability to damage the genetic material of bacteria through the generation of reactive oxygen species, leading to abnormal cell growth and ultimately cell death. Overall, the antibacterial activity studies demonstrate that the synthesized NPs are highly effective at inhibiting bacterial growth. A comparison table of the zone of inhibition (cm) for antibacterial activity of various metal oxides and metal-doped metal oxide NPs, along with the current study and ZOI for penicillin (30 µg ml^−1^; Hi-Media), was used as the positive control for current antibacterial studies as shown in electronic supplementary material, tables S3 and S4.

## 9. Conclusions

A straightforward and cost-effective combustion method was employed to successfully synthesize NiO and Ag/NiO NPs using nickel nitrate and silver nitrate as oxidizers, with coconut water (*C. nucifera*) serving as the fuel at 450°C. XRD analysis confirmed the presence of Ag in the bunsenite form, exhibiting a rhombohedral structure within a simple cubic system, consistent with JCPDS no. 22-1189 for NiO NPs and JCPDS no. 4-783 for metallic Ag. The resulting NPs had an average size of 31−44 nm. XPS and EDX confirmed the successful formation of Ag within the NiO nanostructure. FTIR spectroscopy revealed absorption bands in the range of 460−900 cm^−1^, corresponding to the stretching vibrations of metal–oxygen bonds (Ni–O and Ag–O). A broad FTIR peak at approximately 3454 cm⁻¹ was attributed to the stretching vibrations of O–H molecules in water, while a peak at 1648 cm⁻¹ was associated with the bending vibrations of H–O–H molecules. UV–Vis spectrometry showed reduced band gap energy of Ag/NiO NPs (3.03−2.87 eV) compared to the bare NiO NPs (3.21 eV), indicating a red shift in optical response toward the visible region due to Ag doping. The 0.3 wt% Ag/NiO NPs exhibited the highest quantum efficiency (0.781) among the synthesized samples. PL spectroscopy indicated that a doping concentration of 0.3 wt% Ag effectively introduces donor levels, defect levels and surface trap states within the NiO nanocrystalline structure, enhancing charge carrier separation and reducing recombination. SEM image revealed a voluminous, porous surface morphology characterized by numerous voids, resulting from the release of various combustible gases during the combustion process. This porous structure contributes to the enhanced photocatalytic and antibacterial activities of the 0.3 wt% Ag/NiO NPs. TEM images showed that most particles were spherical, irregular in size and well-distributed, with minimal aggregation occurring during synthesis. The average particle size determined by TEM was 25.8 nm. Raman spectroscopy confirmed the successful doping of Ag into the NiO nanostructure, as well as the high purity and cubic phase of the synthesized NPs. BET analysis of both NiO and 0.3 wt% Ag/NiO NPs exhibited type IV adsorption isotherms, indicating mesoporous structures and a clear monolayer–multilayer adsorption process, 0.3 wt% Ag/NiO NPs showed the highest surface area (170 m^2^ g^−1^) compared to the NiO (130 m^2^ g^−1^) NPs. The Ag/NiO NPs demonstrated a promising H_₂_ evolution rate of 1212 μmol g⁻¹ under visible light illumination in a water/ethanol system. Degradation studies showed up to 98% degradation efficiency and moderate stability for the reusable photocatalysis process. Additionally, the synthesized NPs exhibited significantly enhanced antibacterial activity against various bacterial strains.

## Data Availability

The datasets supporting this article have been uploaded as part of the electronic supplementary material [47]. Data for chemical composition and optical properties for all samples are uploaded as Excel files, text file and synthesis part as video using the Dryad platform [47]. Supplemental information: https://doi.org/10.5281/zenodo.13925417. Supplementary material is available online [48].
